# Supplementary material for: Dispositional goal orientation and perceptions of coach motivational climate on attitudes towards doping among Kenyan endurance runners
Source: PLoS One. 2025 Apr 24;20(4):e0321383. doi: 10.1371/journal.pone.0321383 (PMC12021195; doi:10.1371/journal.pone.0321383)
Supplement: Attachment — Submitted filename: RESPONSE TO REVIEWERS SUBMIT.docx [file pone.0321383.s002.docx]

Kevin K. Kipchumba,

Kenyatta University,

Physical Education and Exercise Science Department,

P. O Box 43844-00100,

Nairobi, Kenya.

Mobile: +254 723 716 230 or +254 708496708

Email: [kevinkipchumba@gmail.com](mailto:kevinkipchumba@gmail.com) or [Kipchumba.kevin@ku.ac.ke](mailto:Kipchumba.kevin@ku.ac.ke)

August 19, 2024

Editor-in-Chief

PLOS

1265 Battery Street, Suite 200

San Francisco, CA 94111

United States

Dear Editor-in-Chief,

**RE: RESPONSE TO REVIEWERS**

I am writing to respond to reviewers and academic editor comments in regards to Research Manuscript ID **PONE-D-24-23784** entitled **“Dispositional Goal Orientation and Perceptions of Coach Motivational Climate on Attitudes towards Doping among Kenyan Endurance Runners”.** We appreciate the insightful comments and suggestions provided by the academic editor and reviewers. We have carefully considered each point raised and have made the necessary revisions to enhance the quality and clarity of our manuscript. Below, we outline our responses to the specific concerns and describe how each has been addressed.

**Response to Academic Editor's Comments**

1. **Enhance the clarity of the research gap and organize the literature review more effectively**

We have revised the Introduction and Literature Review sections to clearly articulate the research gap and ensure a more logical organization of the literature. These changes have enhanced the focus and clarity of the manuscript, making the research gap more explicit.

2. **Explain the reliability and validity of the instruments utilized. Additionally, discuss the justification for selecting non-parametric tests.**

The reliability and validity of the instruments have been addressed by providing a detailed discussion in the Methodology section. Specifically, the reliability of the questionnaires has been supported by Cronbach’s alpha values, which were above the acceptable level of 0.70 for most variables (citing Nunnally, 1978; Shaughnessy et al., 2003).

In order to justify the selection of non-parametric tests, we have cited the results of skewness and kurtosis tests, which indicated that the data was not normally distributed. The skewness values fall below -2 and +2, and kurtosis values fell below -7 and +7, which necessitated the use of non-parametric inferential tests (Hair et al., 2010; Bryne, 2010). These revisions are reflected in the Methods section of our manuscript.

3. **Present statistical values and discuss their practical significance**.

In response to the reviewers' comments, the Results of the study were previously addressed in the Discussion sections. After reading relevant literature and PLOS ONE guidelines, we believe that our manuscript has included the relevant statistical values and more in-depth discussion of their practical significance.

4. **Extend the practical implications and provide specific recommendations.**

The Conclusion section contain a more detailed practical implications and specific recommendations based on our findings offering more actionable insights for researchers, coaches, and sports organizations

5. **Ensure proper formatting of references.**

We have reviewed and corrected the reference list to ensure it meets PLOS ONE's formatting requirements. Unpublished Ph.D. theses have been removed, and all references have been thoroughly checked for accuracy and completeness.

6. **To improve the overall quality of your writing, it may be necessary to re-evaluate the English language standards employed in your manuscript.**

We have revised the manuscript to improve the clarity and quality of the English language. This involved rephrasing sentences for better readability and correcting any grammatical errors.

**Specific Response to Reviewer's Comments:**

1. **Introduction**

We have revised the first line of the introduction to eliminate the repetition and have reviewed the entire section to ensure clarity and coherence. The language has been polished for improved readability.

2. **Method**

We have justified the method used for determining the sample size and provided a more detailed explanation of the procedures followed. This information has been added to the Methods section for greater transparency.

3. **Discussion**

We have removed repetitive content between the Discussion and Results sections and expanded the mechanisms underlying both significant and non-significant results to provide a deeper understanding of the findings.

**Additional Journal-Specific Requirements:**

1. **Data Availability:**

In compliance with the journal’s data policy, we have provided contact information for a non-author institutional body that can respond to data access requests. This information is included in the Contact information.

We have uploaded a revised manuscript with tracked changes, an unmarked clean copy, and this rebuttal letter as separate files. We trust that these revisions address all the concerns raised and hope that the revised manuscript is now suitable for publication in PLOS ONE.

Thank you once again for your constructive feedback and for considering our manuscript for publication.

Yours sincerely,

Kevin Kipchumba

**Kenyatta University, Nairobi, Kenya**
