# Supplementary material for: Dispositional goal orientation and perceptions of coach motivational climate on attitudes towards doping among Kenyan endurance runners
Source: PLoS One. 2025 Apr 24;20(4):e0321383. doi: 10.1371/journal.pone.0321383 (PMC12021195; doi:10.1371/journal.pone.0321383)
Supplement: Attachment — Submitted filename: RESPONSE TO REVIEWERS SUBMIT FEB 2025.docx [file pone.0321383.s004.docx]

Kevin K. Kipchumba,

Kenyatta University,

Physical Education and Exercise Science Department,

P. O Box 43844-00100,

Nairobi, Kenya.

Mobile: +254 723 716 230 or +254 708496708

Email: [kevinkipchumba@gmail.com](mailto:kevinkipchumba@gmail.com) or [Kipchumba.kevin@ku.ac.ke](mailto:Kipchumba.kevin@ku.ac.ke)

February 13, 2025

Editor-in-Chief

PLOS

1265 Battery Street, Suite 200

San Francisco, CA 94111

United States

Dear Editor-in-Chief,

**RE: RESPONSE TO REVIEWERS**

I am writing to respond to reviewers and academic editor comments in regards to Research Manuscript ID **PONE-D-24-23784** entitled **“Dispositional Goal Orientation and Perceptions of Coach Motivational Climate on Attitudes towards Doping among Kenyan Endurance Runners”.** We appreciate the insightful comments and suggestions provided by the academic editor and reviewers. We have carefully considered each point raised and have made the necessary revisions to enhance the quality and clarity of our manuscript. Below, we outline our responses to the specific concerns and describe how each has been addressed.

**Response to Academic Editor's Comments**

1. **Flow of Information: The transition from the literature review to the methodology is abrupt. A concluding paragraph in the literature review would create a smoother transition**

We have revised the Literature Review and methodology sections with concluding paragraph at the end of the literature review section guided by reviewer statements to create smoother transition to methodology.

2. **Coherence Between Paragraphs:**

These has been addressed by linking the two areas in the results section guided by reviewer statements. These revisions are reflected in the Results section of our manuscript.

3. **Clarity:**

These concern from the reviewers have been addressed by rephrasing the sentence and adding guided information from the reviewers to improve on the clarity.

4. **Structure:**

These has been addressed by consolidating the two areas in the results section. These revisions are reflected in the Results section of our manuscript.

5. **Logical Gaps:**

We have revised our manuscript to address logical gaps by discussing the findings’ implications within the context of existing literature and adding guiding statements from the reviewers, thereby strengthening the conclusion and recommendations.

Thank you once again for your constructive feedback and for considering our manuscript for publication.

Yours sincerely,

Kevin Kipchumba

**Kenyatta University, Nairobi, Kenya**
